# *Lepidium graminifolium* L.: Glucosinolate Profile and Antiproliferative Potential of Volatile Isolates

**DOI:** 10.3390/molecules26175183

**Published:** 2021-08-27

**Authors:** Azra Đulović, Franko Burčul, Vedrana Čikeš Čulić, Mirko Ruščić, Petra Brzović, Sabine Montaut, Patrick Rollin, Ivica Blažević

**Affiliations:** 1 Department of Organic Chemistry, Faculty of Chemistry and Technology, University of Split, Ruđera Boškovića 35, 21000 Split, Croatia; azra@ktf-split.hr (A.Đ.); pbrzovic@ktf-split.hr (P.B.); 2 Department of Analytical Chemistry, Faculty of Chemistry and Technology, University of Split, Ruđera Boškovića 35, 21000 Split, Croatia; franko@ktf-split.hr; 3 School of Medicine, University of Split, Šoltanska 2, 21000 Split, Croatia; vedrana.cikes.culic@mefst.hr; 4 Department of Biology, Faculty of Science, University of Split, Ruđera Boškovića 33, 21000 Split, Croatia; mrus@pmfst.hr; 5 Biomolecular Sciences Programme, School of Biological, Chemical and Forensic Sciences, Laurentian University, 935 Ramsey Lake Road, Sudbury, ON P3E 2C6, Canada; smontaut@laurentian.ca; 6 Institut de Chimie Organique et Analytique, Université d’Orléans et CNRS, UMR 7311, 45000 Orléans, France; patrick.rollin@univ-orleans.fr

**Keywords:** *Lepidium graminifolium* L., glucosinolates, desulfoglucosinolates, 3,4,5-trimethoxybenzyl glucosinolate, benzyl isothiocyanate, antiproliferative activity

## Abstract

Glucosinolates (GSLs) from *Lepidium graminifolium* L. were analyzed qualitatively and quantitatively by their desulfo-counterparts using UHPLC-DAD-MS/MS technique and by their volatile breakdown products-isothiocyanates (ITCs) using GC-MS analysis. Thirteen GSLs were identified with arylaliphatic as the major ones in the following order: 3-hydroxybenzyl GSL (glucolepigramin, **7**), benzyl GSL (glucotropaeolin, **9**), 3,4,5-trimethoxybenzyl GSL (**11**), 3-methoxybenzyl GSL (glucolimnanthin, **12**), 4-hydroxy-3,5-dimethoxybenzyl GSL (3,5-dimethoxysinalbin, **8**), 4-hydroxybenzyl GSL (glucosinalbin, **6**), 3,4-dimethoxybenzyl GSL (**10**) and 2-phenylethyl GSL (gluconasturtiin, **13**). GSL breakdown products obtained by hydrodistillation (HD) and CH_2_Cl_2_ extraction after hydrolysis by myrosinase for 24 h (EXT) as well as benzyl ITC were tested for their cytotoxic activity using MTT assay. Generally, EXT showed noticeable antiproliferative activity against human bladder cancer cell line UM-UC-3 and human glioblastoma cell line LN229, and can be considered as moderately active, while IC_50_ of benzyl ITC was 12.3 μg/mL, which can be considered as highly active.

## 1. Introduction

Glucosinolates (GSLs) are sulfur- and nitrogen-containing plant secondary metabolites that can be divided into three classes based on the structure of different amino acid precursors: aliphatic GSLs derived from methionine, isoleucine, leucine or valine, arylaliphatic GSLs derived from phenylalanine or tyrosine, and indole GSLs derived from tryptophan. A recent review in the field of GSLs revealed that only 88 of 137 GSLs found in the plant kingdom were fully characterized by modern spectroscopy techniques up to mid-2018 [1]. More recently, two new GSLs were fully characterized, 4′-*O*-*β*-D-apiofuranosyl-3-hydroxybenzyl GSL (4′-*O*-*β*-D-apiofuranosylglucomatronalin), isolated from *Hesperis laciniata* All. and from *Hesperis matronalis* L. (Brassicaceae) [2] and 2″-*O*-(α-L-arabinopyranosyloxy)benzylglucosinolate (glucoochradenin) from the desert plant *Ochradenus baccatus* Delile (Resedaceae) [3]. An intriguing aspect of this diversity is the recurrence of GSLs derived from glucotropaeolin (benzyl GSL) in wild and cultivated plants such as several species of *Hornungia*, *Lepidium*, *Pentadiplandra*, *Reseda, Sinapis*, *Tropaeolum*, and *Moringa*, which are all characterized by quite different biological activities [4,5,6,7]. These activities are usually correlated to the presence of isothiocyanates (ITCs), but under certain conditions, those can show instability [8]. De Nicola et al. reported that in hydrodistillation-mimicking conditions, most benzylic-type ITCs underwent conversion into the corresponding benzyl alcohols or benzylamines [9].

The genus *Lepidium* (Brassicaceae family) comprises ca. 230 species among which seven are known to be wild-growing in Croatia [10,11]. *Lepidium graminifolium* (grassleaf pepperweed) is a perennial plant widely distributed throughout the Mediterranean region and Middle Europe. It has erect glabrous or sparsely hairy stems up to 50 cm, branched above with small lanceolate leaves, white flowers clustered at the apex, glabrous, ovate-elliptic, scarcely winged fruits and wingless and elliptic seeds [11]. It flowers from May to October. An earlier study by Friis and Kjaer showed the presence of 3-hydroxybenzyl GSL (**7**) and 3-methoxybenzyl GSL (**12**) [12]. Subsequently, analysis of hydrolysis products by GC-MS, the presence of benzyl GSL (**9**), 3-methylsulfanylpropyl GSL [13], **7**, **12**, 4-hydroxybenzyl GSL (**6**), 3,4-dimethoxybenzyl GSL (**10**), and 3,4,5-trimethoxybenzyl GSL (**11**) was reported [14]. Indol-3-ylmethyl GSL, 4-methoxyindol-3-ylmethyl GSL, and 1-methoxyindol-3-ylmethyl GSL were also detected by HPLC analysis of seedlings [15]. A systematic qualitative investigation of the GSL profile of *L. graminifolium* wild-growing in Croatia was performed using LC-MS of intact GSLs, and the major GSL, **7**, was isolated and characterized using spectroscopic techniques [16]. The presence of **6**, **9**, **12** and 4-methoxyindol-3-ylmethyl GSL was confirmed, while the presence of (*2R*)-hydroxybut-3-enyl GSL (**1**), (*2S*)-hydroxybut-3-enyl GSL (**3**), but-3-enyl GSL (**4**), and 2-phenylethyl GSL (**13**) was reported for the first time [16]. However, all previous reports provided no information on the GSL quantity in *L. graminifolium*, especially important due to the presence of non-ubiquitous substituted benzylic-type GSLs.

Thus, the aim of the present work was GSL quantification in different plant parts of wild-growing *L. graminifolium*. GSLs were identified and quantified by their desulfo-counterparts using UHPLC-DAD-MS/MS. The identification of the present GSLs was also performed using GC-MS of the volatiles produced after hydrodistillation and extraction after hydrolysis by myrosinase. Furthermore, the antiproliferative activity of *L. graminifolium* volatiles and benzyl ITC was investigated using the MTT method against human bladder cancer cell line UM-UC-3 and human glioblastoma cell line LN229.

## 2. Results and Discussion

### 2.1. Glucosinolates and Volatile Constituents

**Figure 1:**
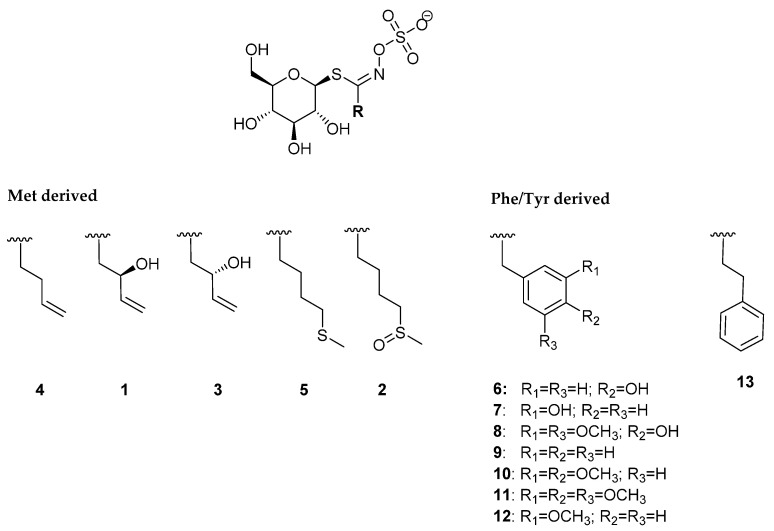
Structures of the GSLs identified in *Lepidium graminifolium* (cf. Table 1).

GSLs of *L. graminifolium* were qualitatively and quantitatively analyzed using UHPLC-DAD-MS/MS by their desulfo-counterparts (Table 1, Figure 1, Figures S1 and S2). The qualitative analysis of GSLs was also confirmed by their breakdown products obtained through enzymatic and/or thermal degradation. Isothiocyanates (ITCs), nitriles and other volatiles originating from GSL degradation were identified by GC-MS. (Table 2).

In total, thirteen GSLs were detected by UHPLC-DAD-MS/MS analyses. The structures of the corresponding GSLs are shown in Figure 1.

Generally, the dominant GSLs were from the arylaliphatic class, biosynthetically originating from Phe/Tyr. In total, eight arylaliphatic GSLs were identified as un-, mono-, di- and tri- substituted ones. In plants, benzyl GSL (**9**) sometimes co-occurs with 4-hydroxybenzyl GSL (**6**) [17,18], suggesting that the biosynthesis may in some cases originate from Phe only, with the hydroxyl group added as a secondary modification [1]. The most abundant peak in all samples analyzed (except root) was observed at t_R_ = 5.12 min with characteristic desulfo-GSL sodium adduct *m*/*z* = 368 (Figures S2 and S3). It was identified as 3-hydroxybenzyl GSL (glucolepigramin) (**7**), with quantity ranging from 3.03 to 75.82 μmol/g of DW (Table 1, Figure 1). The corresponding breakdown products, 3-methoxybenzyl ITC and 3-methoxyphenylacetonitrile, were also detected by GC-MS (Table 2). The major GSL in the root from the Split sample was benzyl GSL (**9**), followed by 3,4,5-trimethoxybenzyl GSL (**11**) and **7**, with 61.69, 35.20 and 20.56 μmol/g of DW, respectively. Highly substituted arylaliphatic GSLs are usually restricted to a few genera (e.g., *Matthiola*, *Lepidium*) [5,16]. To our knowledge, however, **11** was reported only in *Lepidium* species*: L. hyssopifolium* Desv., *L. coronopus* (L.) Al-Shebbaz, *L. sordidum* A. Gray, and *L. densiflorum* Schrad. [4,5,15,17,19,20]. Thus, **11** can be suggested as an important chemotaxonomic marker of *Lepidium* spp. Based on the identification of the rare GSL **11** in *L. coronopus* (syn. *Coronopus squamatus* (Forrsk.) Asch.), Radulović et al. even proposed a change of nomenclature of genus *Coronopus* to genus *Lepidium* [21]. In all Split samples, an abundant peak, observed at t_R_ = 5.59 min, showed a characteristic desulfo-GSL sodium adduct *m*/*z* = 428, which was identified as 4-hydroxy-3,5-dimethoxybenzyl GSL **8** (Table 1, Figures S2 and S3), with 3.04–14.88 μmol/g of DW. Additionally, the breakdown products, 4-hydroxy-3,5-dimethoxyphenylacetonitrile and 4-hydroxy-3,5-dimethoxybenzyl ITC were identified by GC-MS (Table 2, Figure S4). GSL **8** was previously reported only *in L. densiflorum* [4].

Five minor GSLs **1–5** biosynthetically originate from Met. **1, 3** and **4** were found only in the sample collected from Rab Island, while they were absent in Split samples. They were also present in all parts (inflorescence, stem, root, fruit) reported previously [11]. On the other hand, **2** was present only in Split samples in all parts analyzed by their desulfo-GSL and/or its breakdown product 4-(methylsulfinyl)butyl ITC (sulforaphane). Traces of **5** were found in Split root sample only (Table 1 and Table 2). According to the studies of biosynthetic route in the plant model *Arabidopsis thaliana*, Met-derived GSLs start with eight enzymatic steps in order to elongate the chain by two C atoms, followed by core GSL biosynthesis leading to the parent dihomoMet derived GSL, 4-(methylsulfanyl)butyl GSL (**5**). Further sequential secondary modifications of the parent GSL, via 4-(methylsulfinyl)butyl GSL (**2**) and but-3-enyl GSL (**4**) end with 2-hydroxybut-3-enyl GSL (mixture of two stereoisomers, **1** and **3**) [1,22]. In our case the *L. graminifolium* collected in June 2021 (when it starts growing) contained **2** and **5,** while samples collected in October 2016 contained **1** and **3** with traces of **4**, indicating that the time of harvest may display a snapshot of a different step of the GSL biosynthetic pathway in the plant.

**Figure 2:**
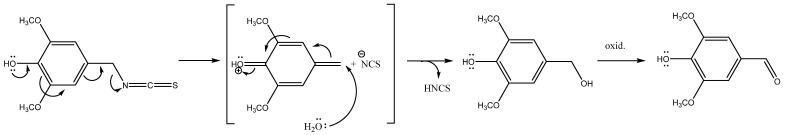
Proposed pathway for the conversion of 4-hydroxy-3,5-dimethoxybenzyl isothiocyanate into the corresponding aldehyde.

Other volatiles included 4-hydroxy-3,5-dimethoxybenzaldehyde and 3-methoxybenzaldehyde, which can be formed due to the instability of the corresponding ITCs during the isolation and/or GC conditions. 4-Hydroxy-3,5-dimethoxybenzaldehyde can be converted from 4-hydroxy-3,5-dimethoxybenzyl alcohol due to the presence of the electron-donating OH group in para position (Figure 2).

### 2.2. Antiproliferative Activity

**Figure 3:**
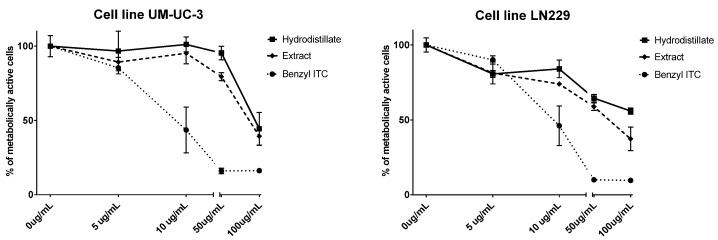
Percentage of metabolically active human bladder cancer cell line UM-UC-3 and human glioblastoma cell line LN229 after 72 h for different concentrations of *L. graminifolium* hydrodistillate, and extract as well as benzyl ITC.

The antiproliferative activity of volatile isolates obtained from Rab Island *L. graminifolium* was tested against human bladder cancer cell line UM-UC-3 and human glioblastoma cell line LN229 (Figure 3) using MTT assay and IC_50_ values were calculated.

According to the IC_50_ values, the antiproliferative activities of hydrodistillate and extract against UM-UC-3 cells after 72 h were ca. 100 μg/mL, which can be considered as moderately active. IC_50_ of benzyl ITC was 12.3 μg/mL, which can be considered as highly active. Based on the GC-MS analysis, it can be suggested that the formation of nitriles in HD (87.41%), mostly phenylacetonitrile (85.72%) from **9** (instead of benzyl ITC, 6.04%), resulted in lower antiproliferative activities. Similarly, EXT contained high percentages of nitriles (48.76%) in comparison to ITCs (5.82%), mostly from **9** and **12** which suggested the same conclusion. However, regulation of cell proliferation, cell cycle, and apoptosis plays crucial roles in the ITC-induced anti-cancer effects, and such phenomena are mainly regulated by complex mechanisms involving caspases, Bcl-2 family proteins, and mitochondrial activities [23]. Benzyl ITC has shown antiproliferative and proapoptotic activity in bladder cells [24,25]. The treatment of UM-UC-3 cells with benzyl ITC and phenylethyl ITC at low micromolar concentrations caused the damage of both outer and inner mitochondrial membranes, leading to the release of cytochrome *c* into the cytoplasm and caspase-9 activation as the major step leading to induction of apoptosis in this cell line [24]. Additionally, benzyl ITC mitochondrial damage is regulated by various members of the Bcl-2 family, including Bcl-2, Bax, Bak, and Bcl-xl [24]. Moreover, Tang et al. found that the urinary *N*-acetylcysteine conjugate of benzyl ITC suppressed different bladder cancer cells’ (RT4, UM-UC-6, UM-UC-6/dox) growth through antiproliferative and proapoptotic activities. The antiproliferative mechanisms of benzyl ITC and its *N*-acetylcysteine conjugate were identical, but relatively longer treatment time or slightly higher doses were needed for the latter compound to exert the same effect [25].

The activity of the extracts against tested LN229 cell line observed in the same time period was similar to the one against UM-UC-3, while HD showed lower activity (Figure 3). The IC_50_ of benzyl ITC was the same as against UM-UC-3 cells, i.e., 12.3 μg/mL, which can be considered as highly active. The activity of benzyl ITC against LN229 was not previously studied, as far as the authors know [26]. Zhu et al. reported that benzyl ITC can inhibit proliferation of human glioma U87MG cells, induce apoptosis and cell cycle arrest of U87MG cells, the mechanism of which may be related to the fact that benzyl ITC can cause oxidative stress to tumor cells [27]. Tang et al. reported that benzyl ITC induced cytotoxic effects through the cell cycle arrest and affected cell cycle-associated gene expression and the induction of cell apoptosis in GBM 8401 cells in vitro [28], while Shang et al. reported benzyl ITC to induce apoptosis of GBM 8401 cells via activation of caspase-8/Bid and the reactive oxygen species-dependent mitochondrial pathway [29]. Phenylethyl ITC, structural analogue of benzyl ITC, was found to inhibit the growth of LN229 cells. It was shown that this ITC can arrest the cell cycle at phase G2/M. Furthermore, it was observed that it can raise ROS expression in the tumor cells, thus suggesting that it can activate caspase-3 activity by affecting the cell cycle and inhibiting the superoxide dismutase activity as well as the glutathione expression [30].

## 3. Materials and Methods

### 3.1. Materials and Reagents

*Lepidium graminifolium* L. samples were obtained from wild-growing plants collected from two locations: Rab Island (43°30ʹ41.8ʹʹ N, 16°28ʹ02.7ʹʹ E; October, 2016) and Split (44°43ʹ53.8ʹʹ N, 14°50ʹ27.9ʹʹ E; June 2021). The botanical identity of the plant material was confirmed by a local botanist, Dr. Mirko Ruščić, from the Faculty of Natural Sciences, University of Split, Croatia and stored under voucher numbers HCPMFST 2030 (old v.n. DBLG001) for Rab sample and HCPMFST 4350 for Split sample. Myrosinase, benzyl ITC and sinigrin were obtained from Sigma Aldrich (St. Louis, MO, USA). All other chemicals and reagents were of analytical grade. Cancer cell lines (human bladder cancer cell line UM-UC-3 and human glioblastoma cell line LN229) were cultured in a humidified atmosphere with 5% CO_2_ at 37 °C, in Dulbecco’s modified Eagle medium (DMEM, EuroClone, Milan, Italy) containing 4.5 g/L glucose, 10% fetal bovine serum (FBS) and 1% antibiotics (penicillin streptomycin, EuroClone, Milan, Italy).

### 3.2. Isolation and Chemical Analysis

#### 3.2.1. Isolation of Desulfoglucosinolates

GSLs were extracted as previously reported [31,32]. The dried plant parts of Rab sample (aerial part) and Split sample (flower, leaf, stem, siliquae, root) were ground to a fine powder, from which 100 mg were extracted for 5 min at 80 °C in 2 × 1 mL MeOH/H_2_O (70:30 *v*/*v*) to inactivate the endogenous myrosinase. Each extract (1 mL) was loaded onto a mini-column filled with 0.5 mL of DEAE-Sephadex A-25 anion-exchange resin (GE Healthcare, Chicago, IL, USA) conditioned with 25 mM acetate buffer (pH 5.6). After washing the column with 70% MeOH and 1 mL of ultrapure water, optimal conditions for desulfation were set by adding buffer solution. Each mini-column was loaded with 20 μL (0.35 U/mL) of purified sulfatase and left to stand 18h at room temperature. The desulfo-GSLs were then eluted with 1.5 mL of ultra-pure H_2_O, lyophilized and diluted to the 1 mL. The samples were stored at –20 °C until further analysis by UHPLC-DAD-MS/MS.

#### 3.2.2. UHPLC-DAD-MS/MS Analysis

Analysis was performed on UHPLC-DAD-MS/MS (Ultimate 3000RS with TSQ Quantis MS/MS detector, Thermo Fischer Scientific, Waltham, MA, USA) using Hypersil GOLD column (3.0 µm, 3.0 × 100 mm, Thermo Fischer Scientific). A gradient consisting of solvent A (50 μM NaCl in H_2_O) and solvent B (acetonitrile:H_2_O 30:70 *v*/*v*) was applied at a flow rate of 0.5 mL/min as follows: 0.14 min 96% A and 4% B; 7.84 min 14% A and 86% B; 8.96 min 14% A and 86% B; 9.52 min 5% A and 95% B; 13.16 min 5% A and 95% B; 13.44 min 96% A and 4% B; 15.68 min 96% A and 4% B. The column temperature was held at 25 °C and the injection volume was 5 µL. The electrospray interface was H-ESI source operating with a capillary voltage of 3.5 kV at 350 °C. The system was operated in the positive ion electrospray mode.

The amount of GSLs was quantified using a calibration curve (y = 0.0206x + 0.2371, R^2^ = 0.9992, LOD = 1.67 μM, LOQ = 5.03 μM) of pure desulfosinigrin solution (range from 13.63 to 545.00 µM) and RPFs for each individual desulfo-GSL [33]. RPF values for quantification of desulfo-GSLs were as follows: RPF 1.09 for **1** and **3**, 1.07 for **2**, 1.11 for **4**, 1.04 for **5**, 0.50 for **6**, 0.95 for **9** and **13** [34]; RPF 0.55 for **12** [7]; arbitrary RPF 1.0 for **7**, **8**, **10** and **11**.

#### 3.2.3. Isolation of Volatiles

The volatiles from the Rab sample (aerial part) were isolated by two approaches. Hydrodistillation was performed in Clevenger-type apparatus for 2.5 h using 50 g of dry material (HD). Dry plant material (10 g) was ground (in a coffee grinder), stirred with 20 mL of distilled water, and after endogenous and exogenous hydrolysis by myrosinase (1–2 units) for 24 h at 27 °C and pH~5.4, was extracted using CH_2_Cl_2_ (EXT). pH was determined using a pH meter (Hanna Instruments, Woonsocket, RI, USA). The Split samples (flower, leaf, stem, silliquae, root) were extracted using 1 g of each plant part (EXT) [31,32].

#### 3.2.4. GC-MS Analysis

The gas chromatography system used consisted of gas chromatograph, model 8890 GC, equipped with an automatic liquid injector, model 7693A, and tandem mass spectrometer (MS/MS), model 7000D GC/TQ (Agilent Inc., Santa Clara, CA, USA). The samples were analyzed on a non-polar HP-5MS UI column (dimensions: 30 m length, inner diameter 0.25 mm and stationary phase layer thickness 0.25 μm, Agilent Inc., Santa Clara, CA, USA). The column temperature program was set at 60 °C for the first 3 min and then heated to 246 °C at 3 °C/min, and maintained for 25 min isothermally. The carrier gas was helium, and the flow rate was 1 mL/min. The inlet temperature was 250 °C, while the volume of the injected sample was 1 μL. Other conditions were as follows: ionization energy was 70 eV; ion source temperature was 230 °C; the temperature of the quadrupoles was set at 150 °C. The analyses were carried out in duplicate.

The individual peaks were identified by comparison of their retention indices (relative to C_8_–C_40_ *n*-alkanes for HP-5MS UI column) to those from a homemade library, literature and/or authentic samples, as well as by comparing their mass spectra with literature, Wiley 9N08 MS (Wiley, New York, NY, USA) and NIST17 (Gaithersburg, MD, USA) mass spectral databases. The percentages in Table 2 were calculated as the mean value of component percentages on HP-5MS UI column for analyses run in duplicate.

### 3.3. Cell Viability Assay (MTT)

MTT spectrophotometric assay was performed on a microplate photometer, model HiPo MPP-96 (BioSan, Riga, Latvia) as previously described [19,20]. The criteria used to categorize the activity against the tested cell lines were based on IC_50_ values as follows: 20 μg/mL = highly active, 21–200 μg/mL = moderately active, 201–500 μg/mL = weakly active, and >501 μg/mL = inactive [35]. Thus, the cells were treated with Rab *L. graminifolium* volatile isolates (HD, EXT) at concentrations of 5, 10, 50 and 100 µg/mL in a complete medium (in triplicate) for 72 h. After treatment with isolated compounds, the cells were incubated with 0.5 g MTT/L at 37 °C for 2 h, the medium was removed, then DMSO was added, and the mixture was incubated for another 10 min at 37 °C while shaking. The degree of formazan formation, an indicator of living and metabolically active cells, was measured at 570 nm. The data were calculated in relation to the untreated control (100%) from three independent measurements. The calculation of IC_50_ values was performed using GraphPad Prism software version 7.0.

### 3.4. Statistical Analysis

Analysis of variance (one-way ANOVA) was used to assess the statistical difference between data reported in Table 1, followed by a least significance difference test to evaluate differences between sets of mean values at significance level set at *p* < 0.05. Analyses were carried out using Statgraphics Centurion-Ver.16.1.11 (StatPoint Technologies, Inc., Warrenton, VA, USA) [36].

## 4. Conclusions

GSLs in *L. graminifolium* were quantified for the first time by UHPLC-DAD-MS/MS. In addition, new GSLs were identified in this species: glucoraphanin (**2**)**,** glucoerucin (**5**) and one multi-substituted benzyl GSL, 4-hydroxy-3,5-dimethoxybenzyl GSL (**8**), which was previously reported only in *L. densiflorum*. Multi-substituted benzyl GSLs, such as 3,4,5-trimethoxybenzyl GSL, are rarely found but seem to be common for *Lepidium* species. Generally, the biosynthetic pathways are still poorly investigated, especially in most non-model plants, and they should be the focus of further studies. Antiproliferative effects of the tested volatile isolates rich in GSL breakdown products (mostly nitriles) on cancer cells showed moderate potential, while benzyl ITC showed high potential. Thus, different factors influencing the formation of GSL breakdown products can consequently lead to better activity of isolates containing ITCs. In addition, further experiments on benzylic-type ITCs should be performed to clarify the antiproliferative effects and underlying mechanisms.

## Figures and Tables

**Table 1:** Glucosinolate (GSL) content in *L. graminifolium* diverse plant parts, collected from different locations.

Glucosinolate (GSL) (Trivial Name)			[M + Na]^+^	Glucosinolates µmol/g DW					
				Rab Island	Split				
				Arial Part	Flower	Leaf	Stem	Siliquae	Root
*Met derived*									
	**1**	(*2R*)-Hydroxybut-3-enyl GSL (progoitrin)	332	0.26 ± 0.02 ^A^	-	-	-	-	-
	**2**	4-(Methylsulfinyl)butyl GSL (glucoraphanin)	380	-	7.32 ± 1.67 ^A, V^	3.49 ± 0.34 ^A, W^	-	4.00 ± 0.50 ^A, X^	-
	**3**	(*2S*)-Hydroxybut-3-enyl GSL (epiprogoitrin)	332	1.56 ± 0.11 ^B^	-	-	-	-	-
	**4**	But-3-enyl GSL (gluconapin)	316	tr	-	-	-	-	-
	**5**	4-(Methylsulfanyl)butyl GSL (glucoerucin)	364	-	-	-	-	-	tr
*Phe/Tyr derived*	**6**	4-Hydroxybenzyl GSL (glucosinalbin)	368	0.19 ± 0.06 ^A, D, V^	2.15 ± 0.33 ^B, W^	tr	tr	3.92 ± 0.38 ^A, X^	1.62 ± 0.20 ^A, Y^
	**7**	3-Hydroxybenzyl GSL (glucolepigramin)	368	3.03 ± 0.23 ^C, V^	68.60 ± 7.30 ^C, W^	19.30 ± 0.20 ^B, X^	6.43 ± 1.33 ^A, V^	75.82 ± 8.06 ^B, W^	20.56 ± 1.78 ^B, X^
	**8**	4-Hydroxy-3,5-dimethoxybenzyl GSL (3,5-dimethoxysinalbin)	428	tr	11.12 ± 1.86 ^A, D, V^	14.88 ± 0.05 ^C, W^	3.04 ± 0.85 ^B, X^	12.04 ± 0.21 ^C, V^	12.49 ± 1.48 ^C, V^
	**9**	Benzyl GSL (glucotropaeolin)	352	0.12 ± 0.00 ^D, V^	1.80 ± 0.44 ^B, V, W^	tr	0.47 ± 0.08 ^C, V^	5.14 ± 0.23 ^A, W^	61.69 ± 5.82 ^D, X^
	**10**	3,4-Dimethoxybenzyl GSL	412	tr	-	-	-	-	tr
	**11**	3,4,5-Trimethoxybenzyl GSL	442	tr	13.17 ± 2.64 ^D, V^	3.42 ± 0.08 ^A, W^	0.87 ± 0.00 ^C, X^	11.03 ± 0.31 ^C, V^	35.20 ± 4.33 ^E, Y^
	**12**	3-Methoxybenzyl GSL (glucolimnanthin)	382	1.24 ± 0.10 ^E, V^	-	-	tr	11.95 ± 0.29 ^C, W^	18.18 ± 1.51 ^B, X^
	**13**	2-Phenylethyl GSL (gluconasturtiin)	366	tr	-	-	-	-	-
		Total (µmol/g DW)		6.40 ± 0.52 ^V^	104.16 ± 14.24 ^W^	41.09 ± 0.67 ^X^	10.81 ± 2.26 ^V^	123.90 ± 9.98 ^Y^	149.74 ± 15.12 ^Z^

All MS^2^ spectra are given in Figure S3. Tr-traces. Data are expressed as the mean value ± standard error (*n* = 3). Different superscript letters ^A^^–E^ in the same column denote statistically significant difference (*p* < 0.05) in each glucosinolate content within plant sample/part, while different superscript letters ^V^^–Z^ in the same row denote statistically significant difference (*p* < 0.05) in individual glucosinolate content between plant samples/parts.

**Table 2:** Volatiles obtained from aerial parts of *L. graminifolium* using different methods of isolation.

	Parent Glucosinolate Identified Breakdown Compound	RI	Rab Island		Split			
			HD	EXT	EXT			
			Aerial Part	Aerial Part	Leaf	Stem	Siliquae	Root
*Met derived*	4-(Methylsulfinyl)butyl GSL (glucoraphanin, **2**) 5-(Methylsulfinyl)pentanenitrile ^a, b^ 4-(Methylsulfinyl)butyl ITC (sulforaphane) ^a, b^							
		1512	-	-	1.68	2.34	-	-
		1760	-	-	71.74	52.70	30.34	0.83
	4-(Methylsulfanyl)butyl GSL (glucoerucin, **5**)							
	4-(Methylsulfanyl)butyl ITC (erucin) ^a, b^	1433	-	-	-	-	-	2.29
*Phe/Tyr derived*	3-Hydroxybenzyl GSL (glucolepigramin, **7**)							
	3-Hydroxyphenylacetonitrile ^b, c^	1483	-	-	tr	4.71	-	-
	3-Hydroxybenzyl ITC ^b, c^	1701	-	-	3.19	5.14	20.15	8.61
	4-Hydroxy-3,5-dimethoxybenzyl GSL (3,5-dimethoxysinalbin, **8**)							
	4-Hydroxy-3,5- dimethoxyphenylacetonitrile^c^	1790	-	-	2.94	8.20	-	-
	4-Hydroxy-3,5-dimethoxybenzyl ITC ^c^	2017	-	-	0.98	-	0.43	-
	Benzyl GSL (glucotropaeolin, **9**)							
	Phenylacetonitrile ^a, b^	1141	85.72	18.69	tr	2.52	-	0.31
	Benzyl ITC ^a, b^	1365	6.04	0.53	-	-	tr	27.88
	3,4-Dimethoxybenzyl GSL (**10**)							
	3,4-Dimethoxybenzyl ITC ^b, c^	1822	-	-	-	-	-	tr
	3,4,5-Trimethoxybenzyl GSL (**11**)							
	3,4,5-Trimethoxybenzyl ITC ^b, c^	1961	-	-	1.80	3.06	4.95	18.71
	3-Methoxybenzyl GSL (glucolimnanthin, **12**)							
	3-Methoxyphenylacetonitrile ^b, c^	1377	1.69	29.05	-	0.96	-	tr
	3-Methoxybenzyl ITC ^b, c^	1601	1.07	3.83	-	-	0.90	13.92
	2-Phenylethyl GSL (gluconasturtiin, **13**)							
	3-Phenylpropanenitrile ^a, b^	1242	-	1.02	-	-	-	-
	2-Phenylethyl ITC ^a, b^	1466	tr	1.46	-	-	-	-
	**Other volatiles**							
	Benzaldehyde ^a, b^	963	1.97	-	tr	0.71	0.53	0.32
	Benzyl alcohol ^a, b^	1036	-	-	-	0.96	0.80	0.58
	3-Methoxybenzaldehyde ^b^	1198	-	4.57	-	-	-	-
	2-Methoxy-4-allylphenol (eugenol) ^a, b^	1358	-	-	1.60	8.28	8.04	5.48
	β-Selinene ^a, b^	1486	-	14.16	-	-	-	-
	4-Hydroxy-3,5-dimethoxybenzaldehyde^b^	1659	-	-	1.44	1.70	-	tr
	Caffeine ^a, b^	1839	-	-	7.96	8.20	30.55	18.71
	6,10,14-Trimethylpentadecan-2-one ^a, b^	1846	-	17.46	-	-	-	-
	Hexadecanoic acid ^a, b^	1964	-	5.67	-	-	-	-
	Ethylhexadecanoate ^a, b^	1995	-	1.87	-	-	-	-
	Total (%)		96.49	98.31	93.33	99.48	96.69	97.64

HD, hydrodistillation in Clevenger-type apparatus; EXT, CH_2_Cl_2_ extraction after 24 h of autolysis and added myrosinase; RI, retention indices determined on a HP-5MS UI capillary column; -, not detected; tr, traces; ITC, isothiocyanate. ^a^ Compound identified by mass spectra and RI comparison with homemade library. ^b^ Compound identified by mass spectra comparison with Wiley/NIST library. ^c^ Compound identified by mass spectra comparison with literature values [4]. Spectra are given in Figure S4.

## Supplementary Materials

The following are available online. Figure S1. Chromatogram of desulfo-GSLs obtained from the aerial parts of *L. graminifolium* from Rab Island: d1‒(2*R*)-Hydroxybut-3-enyl GSL (progoitrin), d3‒(2*S*)-Hydroxybut-3-enyl GSL (epiprogoitrin), d4‒But-3-enyl GSL (gluconapin), d6‒4-Hydroxybenzyl GSL (glucosinalbin), d7‒3-Hydroxybenzyl GSL (glucolepigramin), d8‒4-Hydroxy-3,5-dimethoxybenzyl GSL (3,5-dimethoxysinalbin), d9‒Benzyl GSL (glucotropaeolin), d10‒3,4-Dimethoxybenzyl GSL, d11‒3,4,5-Trimethoxybenzyl GSL, d12‒3-Methoxybenzyl GSL (glucolimnanthin), d13‒2-Phenylethyl GSL (gluconasturtiin).; Figure S2. Chromatogram of desulfo-GSLs obtained from the leaf, siliquae, flower, stem and root of *L. graminifolium* from Split: d2‒(2*S*)-Hydroxybut-3-enyl GSL (glucoraphanin), d5‒4-(Methylsulfanyl)butyl GSL (glucoerucin), d6‒4-Hydroxybenzyl GSL (glucosinalbin), d7‒3-Hydroxybenzyl GSL (glucolepigramin), d8‒4-Hydroxy-3,5-dimethoxybenzyl GSL (3,5-dimethoxysinalbin), d9‒Benzyl GSL (glucotropaeolin), d10‒3,4-Dimethoxybenzyl GSL, d11‒3,4,5-Trimethoxybenzyl GSL, d12‒3-Methoxybenzyl GSL (glucolimnanthin).; Figure S3. MS^2^ spectra at 15V ionization of desulfo-GLSs detected: d1- d13.; Figure S4. MS spectra of degradation products originating from substituted benzylic-type GSLs. Numbers correspond to Table 1 and Figure 1.
