# Supplementary material for: Lepidium graminifolium L.: Glucosinolate Profile and Antiproliferative Potential of Volatile Isolates
Source: Molecules. 2021 Aug 27;26(17):5183. doi: 10.3390/molecules26175183 (PMC8434519; doi:10.3390/molecules26175183)
Supplement: Supplementary file 1 [file molecules-26-05183-s001.zip › Blazevic_supplementary.pdf]

molecules
MDPI

Supplementary Materials

Lepidium graminifolium L.: Glucosinolate Profile and Antiproliferative Potential of Volatile Isolates

Azra Đulović[1a], Franko Burčul[1b], Vedrana Čikeš Čulić[2], Mirko Ruščić[3], Petra Brzović[1a], Sabine Montaut[4], Patrick Rollin[5], Ivica Blažević[1a*]

[1] University of Split, Faculty of Chemistry and Technology, a) Department of Organic Chemistry, b) Department of Analytical Chemistry, Ruđera Boškovića 35, 21000 Split, Croatia; blazevic@ktf-split.hr (I.B.); azra@ktf-split.hr (A.Đ.); franko@ktf-split.hr (F.B.)
[2] University of Split, School of Medicine, Šoltanska 2, 21000 Split, Croatia; vedrana.cikes.culic@mefst.hr
[3] University of Split, Faculty of Science, Department of Biology, Ruđera Boškovića 33, 21000 Split, Croatia; mrus@pmfst.hr
[4] School of Biological, Chemical and Forensic Sciences, Biomolecular Sciences Programme, Laurentian University, 935 Ramsey Lake Road, Sudbury, ON, P3E 2C6, Canada ; smontaut@laurentian.ca
[5] ICOA, Université d'Orléans et CNRS, UMR 7311, Orléans, France; patrick.rollin@univ-orleans.fr
* Correspondence: blazevic@ktf-split.hr; Tel.:+385 21 329 434

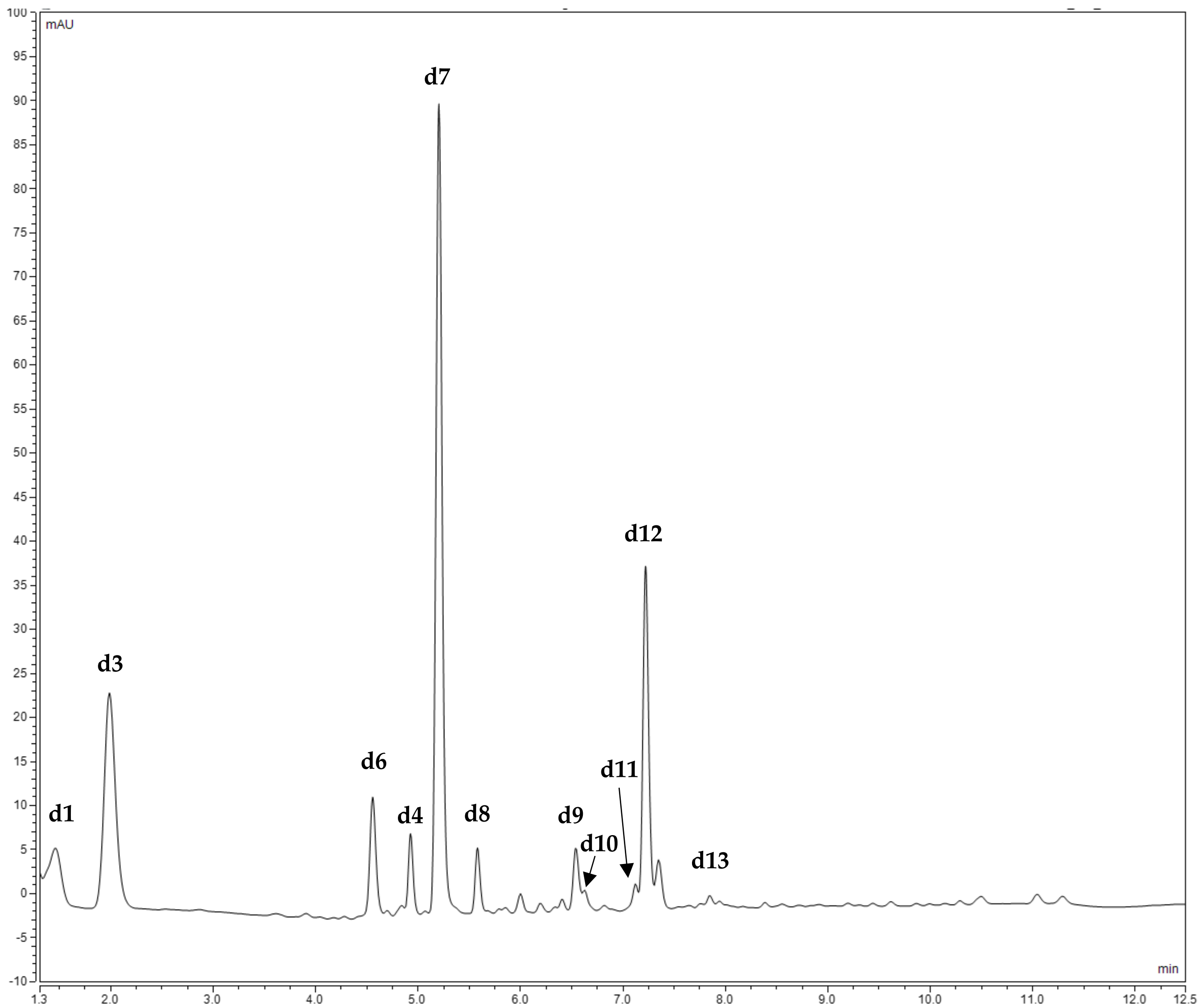

Figure S1. Chromatogram of desulfo-GSLs obtained from the aerial parts of *L. graminifolium* from Rab Island:
d1 - (2*R*)-Hydroxybut-3-enyl GSL (progoitrin), **d3** - (2*S*)-Hydroxybut-3-enyl GSL (epiprogoitrin), **d4** - But-3-enyl GSL (gluconapin), **d6** - 4-Hydroxybenzyl GSL (glucosinalbin), **d7** - 3-Hydroxybenzyl GSL (glucolepigramin), **d8** - 4-Hydroxy-3,5-dimethoxybenzyl GSL (3,5-dimethoxysinalbin), **d9** - Benzyl GSL (glucotropaeolin), **d10** - 3,4-Dimethoxybenzyl GSL, **d11** - 3,4,5-Trimethoxybenzyl GSL, **d12** - 3-Methoxybenzyl GSL (glucolimnanthin), **d13** - 2-Phenylethyl GSL (gluconasturtiin).

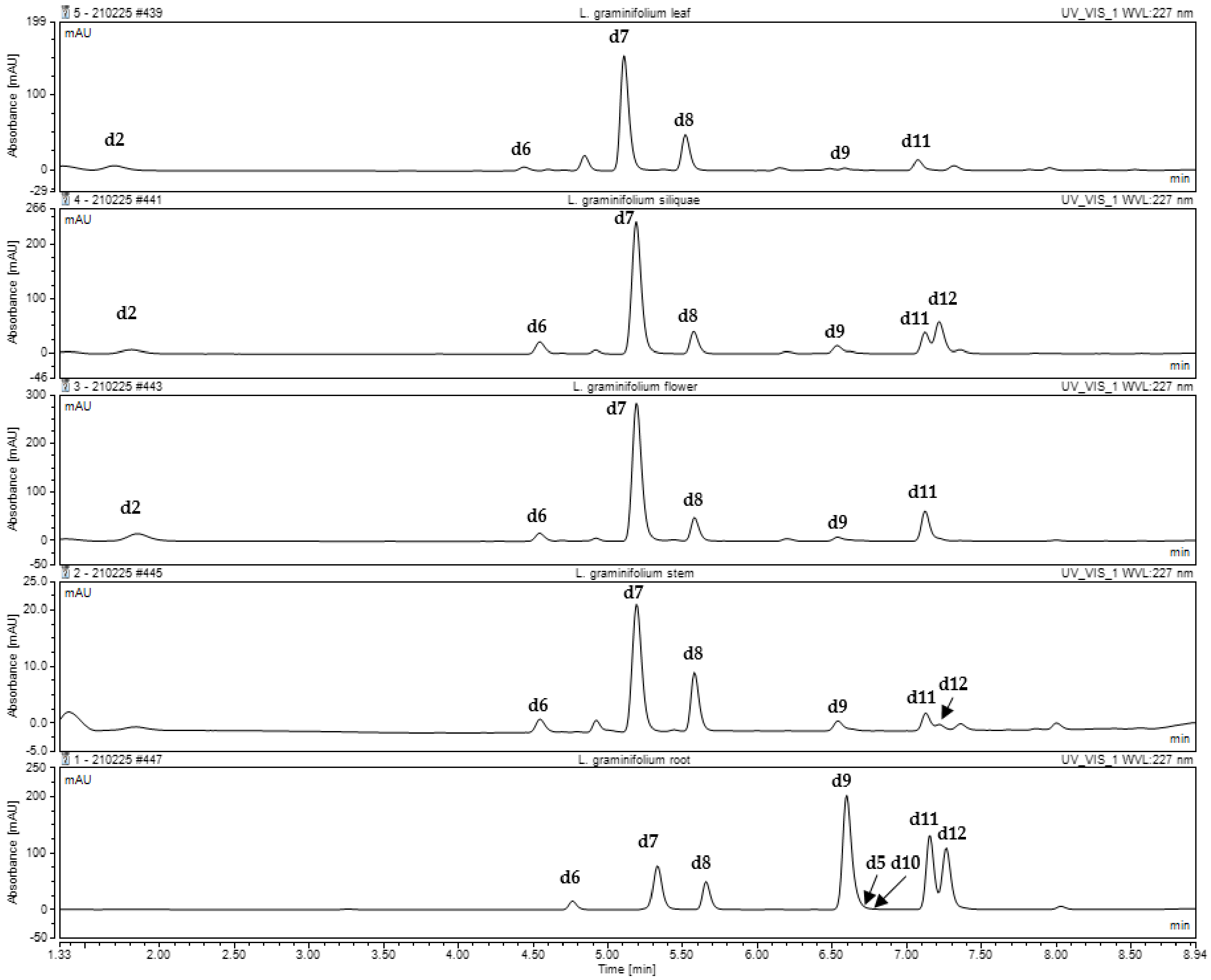

Figure S2. Chromatogram of desulfo-GSLs obtained from the leaf, siliquae, flower, stem and root of *L. graminifolium* from Split: **d2** - (2*S*)-Hydroxybut-3-enyl GSL (glucoraphanin), **d5** - 4-(Methylsulfanyl)butyl GSL (glucoerucin), **d6** - 4-Hydroxybenzyl GSL (glucosinalbin), **d7** - 3-Hydroxybenzyl GSL (glucolepigramin), **d8** - 4-Hydroxy-3,5-dimethoxybenzyl GSL (3,5-dimethoxysinalbin), **d9** - Benzyl GSL (glucotropaeolin), **d10** - 3,4-Dimethoxybenzyl GSL, **d11** - 3,4,5-Trimethoxybenzyl GSL, **d12** - 3-Methoxybenzyl GSL (glucolimnanthin).

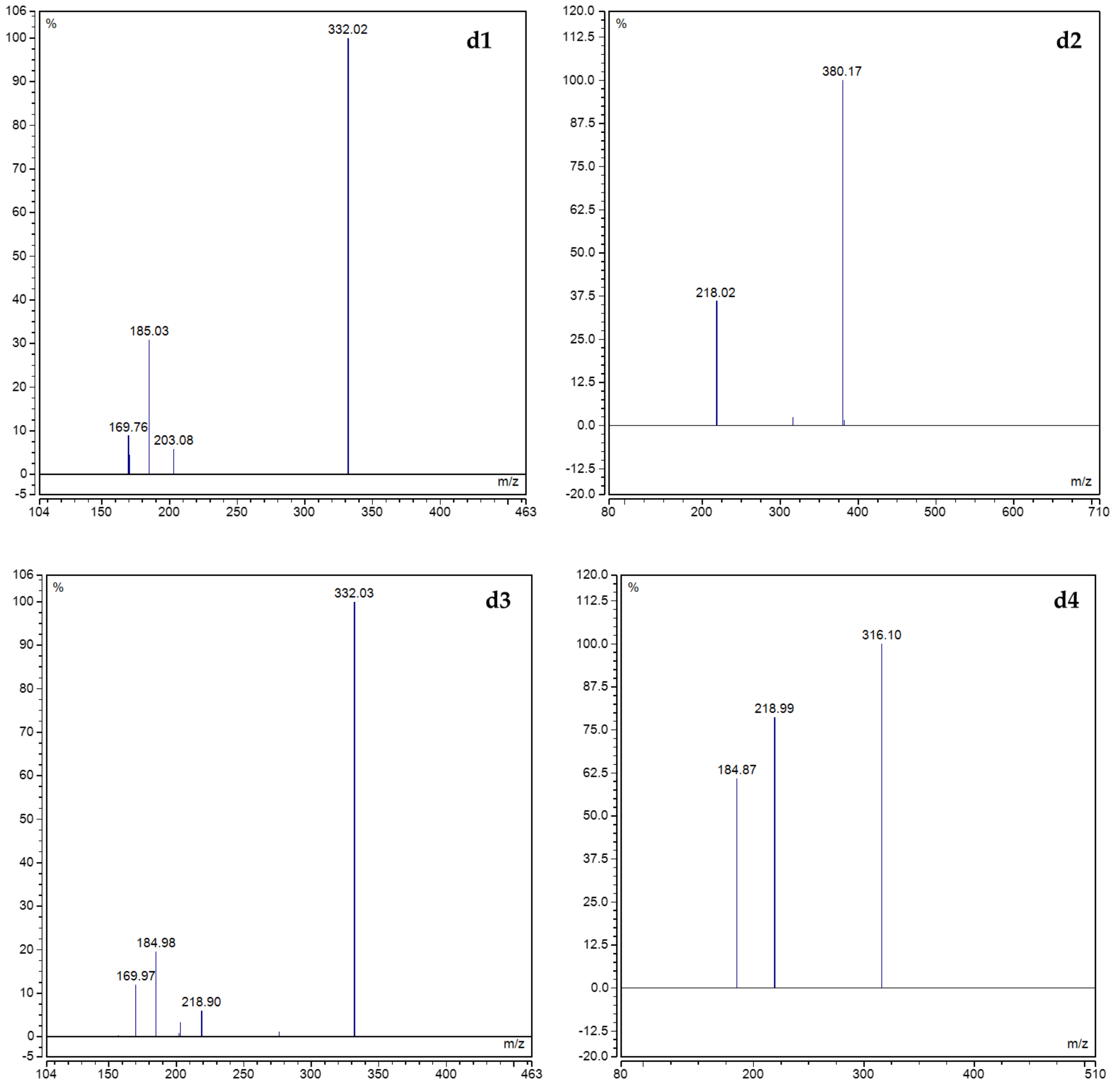

Figure S3. *Continuous*

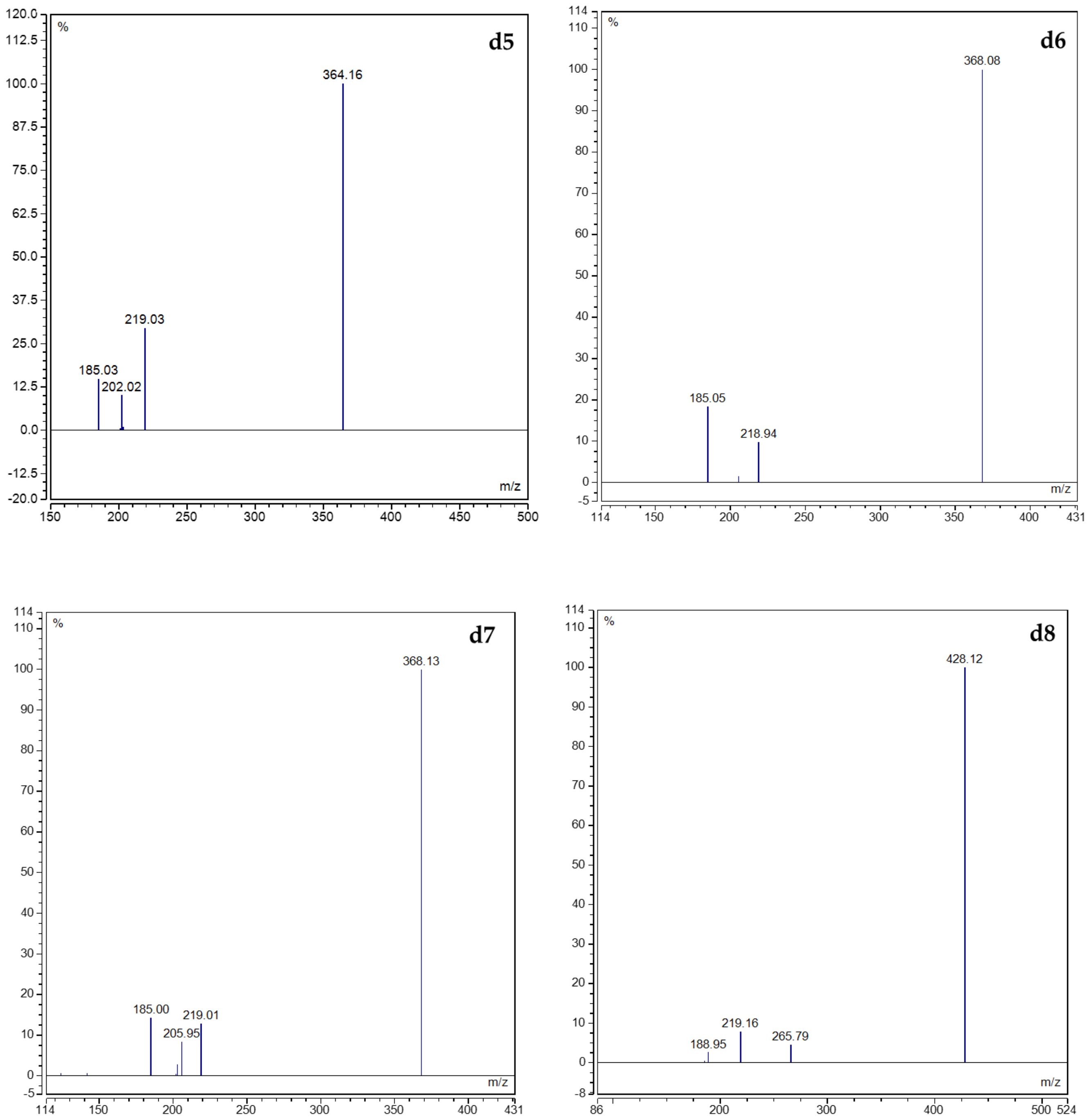

Figure S3. *Continuous*

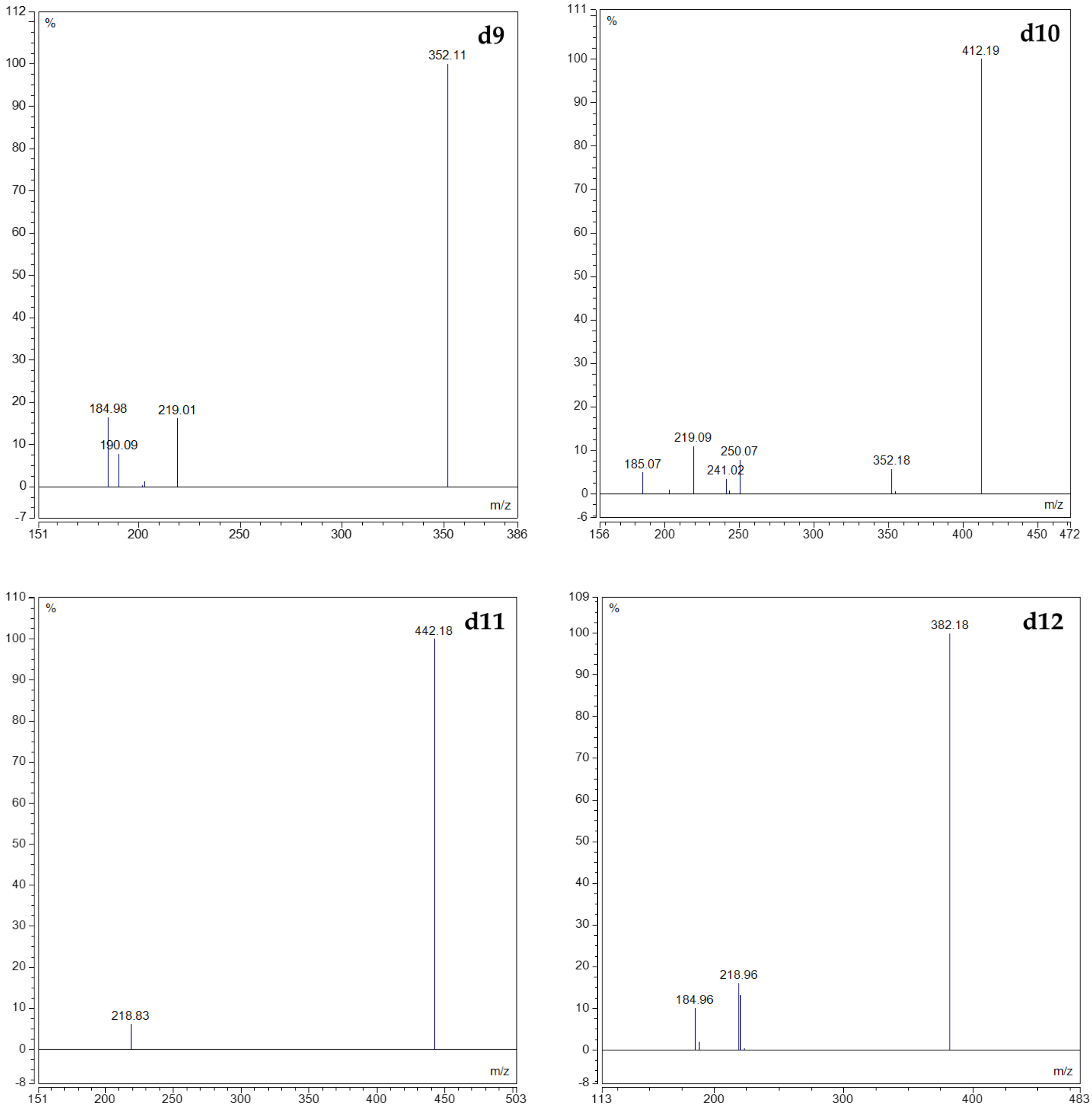

Figure S3. *Continuous*

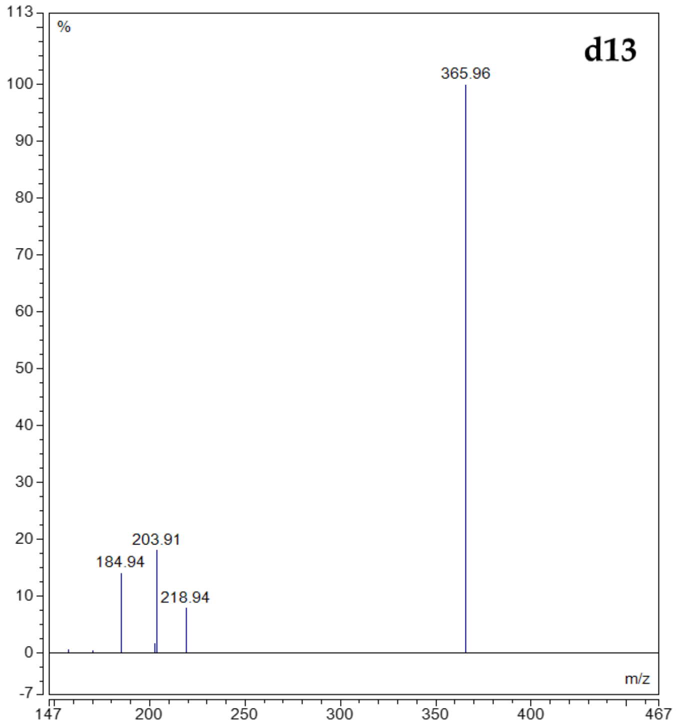

Figure S3. MS² spectra at 15V ionization of desulfo-GSLs detected: **d1**- **d13**.

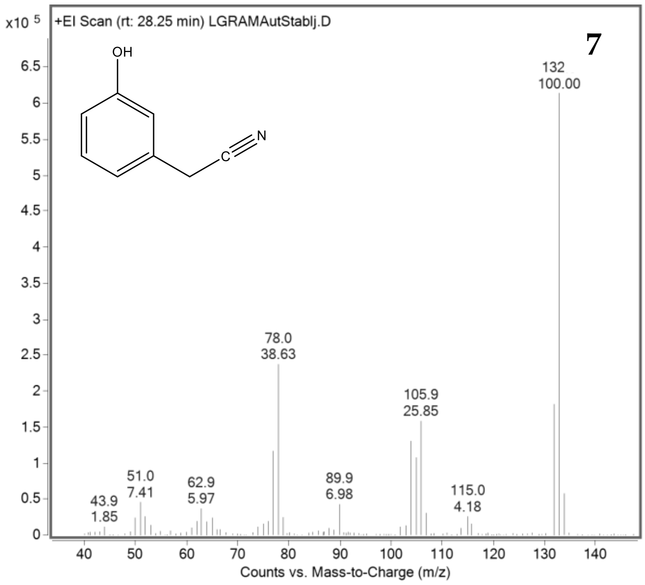
x10 5
+EI Scan (rt: 28.25 min) LGRAMAutStablj.D
7
OH
C
N
132
100.00
78.0
38.63
105.9
25.85
51.0
7.41
62.9
5.97
89.9
6.98
115.0
4.18
43.9
1.85
Counts vs. Mass-to-Charge (m/z)

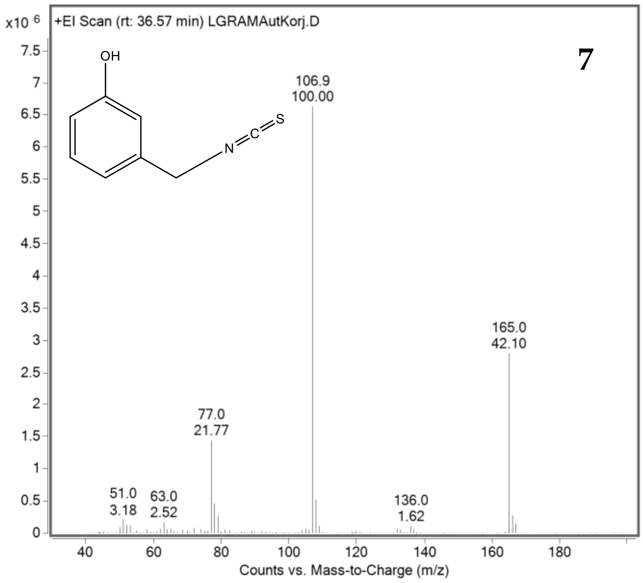
x10 6
+EI Scan (rt: 36.57 min) LGRAMAutKorj.D
7
OH
N
C
S
106.9
100.00
165.0
42.10
77.0
21.77
51.0
3.18
63.0
2.52
136.0
1.62
Counts vs. Mass-to-Charge (m/z)

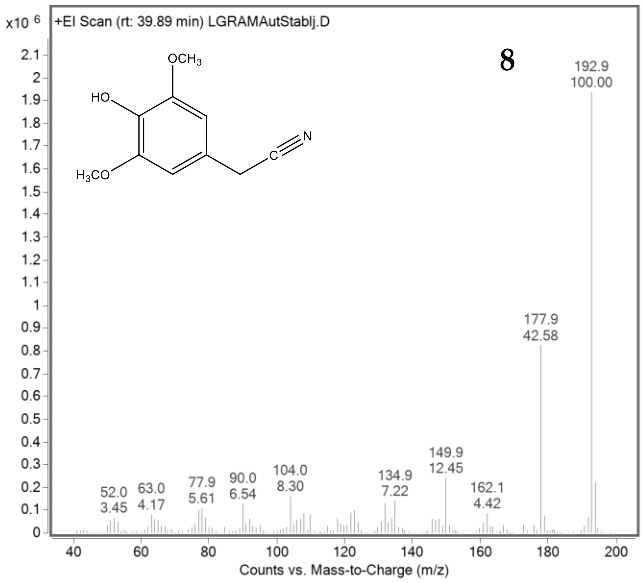
x10 6
+EI Scan (rt: 39.89 min) LGRAMAutStablj.D
8
OCH_3
HO
H_3CO
C
N
192.9
100.00
177.9
42.58
52.0
3.45
63.0
4.17
77.9
5.61
90.0
6.54
104.0
8.30
134.9
7.22
149.9
12.45
162.1
4.42
Counts vs. Mass-to-Charge (m/z)

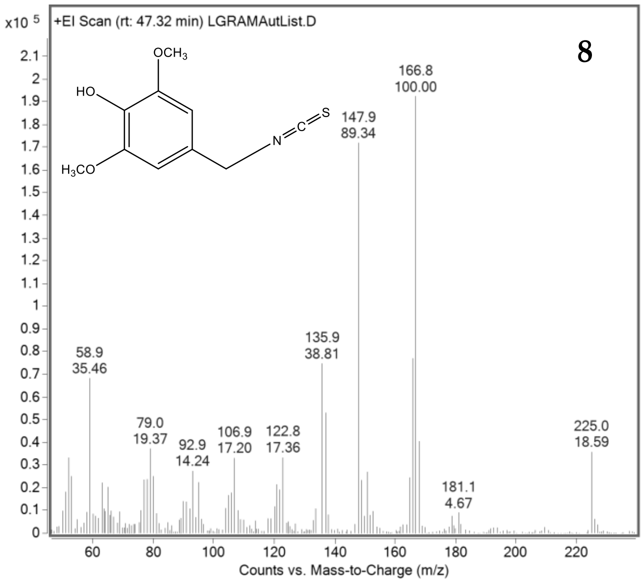
x10 5
+EI Scan (rt: 47.32 min) LGRAMAutList.D
8
OCH_3
HO
H_3CO
N
C
S
166.8
100.00
147.9
89.34
135.9
38.81
58.9
35.46
79.0
19.37
92.9
14.24
106.9
17.20
122.8
17.36
181.1
4.67
225.0
18.59
Counts vs. Mass-to-Charge (m/z)

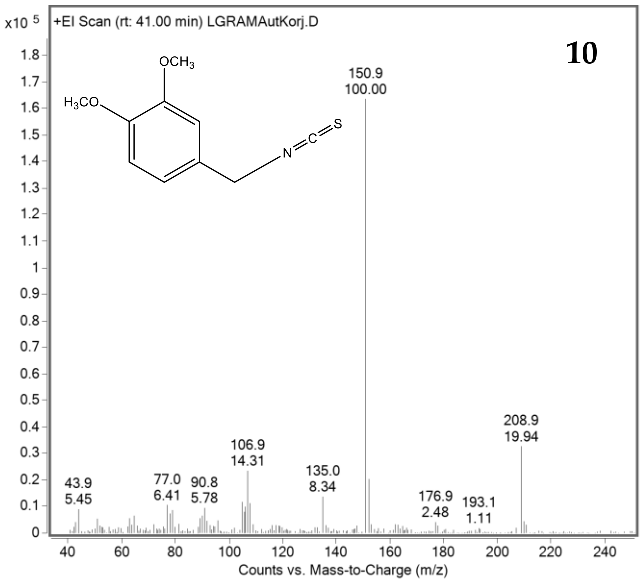
x10 5
+EI Scan (rt: 41.00 min) LGRAMAutKorj.D
10
OCH_3
H_3CO
N
C
S
150.9
100.00
208.9
19.94
106.9
14.31
135.0
8.34
43.9
5.45
77.0
6.41
90.8
5.78
176.9
2.48
193.1
1.11
Counts vs. Mass-to-Charge (m/z)

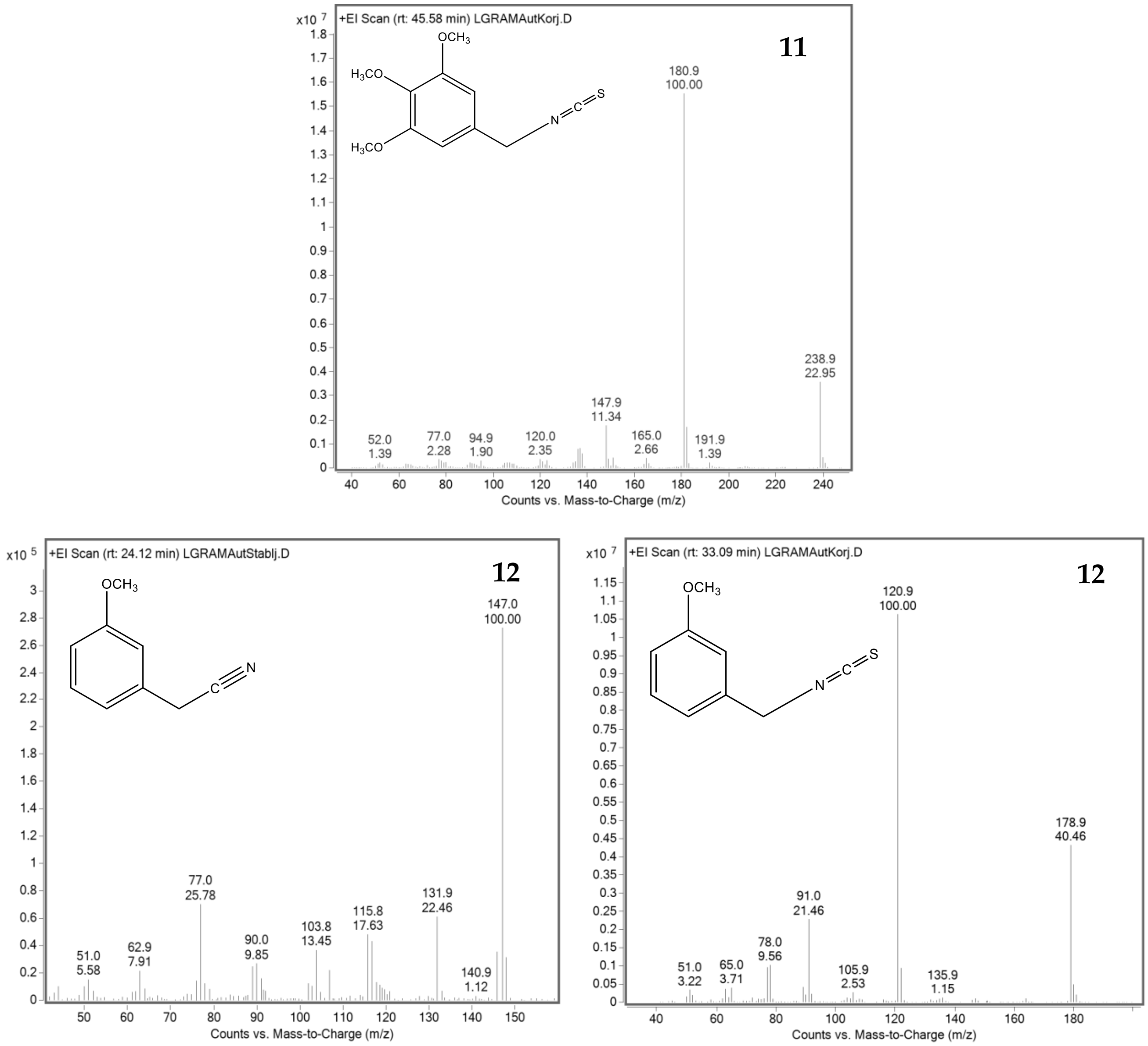

Figure S4. MS spectra of degradation products originating from substituted benzylic-type GSLs. Numbers correspond to Table 1 and Figure 1.
